# On the Reflexive KENDİ in Turkish Sign Language

**DOI:** 10.3389/fpsyg.2022.753455

**Published:** 2022-04-26

**Authors:** Demet Kayabaşı, Natasha Abner

**Affiliations:** Department of Linguistics, University of Michigan, Ann Arbor, MI, United States

**Keywords:** Turkish Sign Language (TİD), reflexives, emphatic reflexives, co-referential relations, anaphora, typology, sign languages, syntax

## Abstract

Linguistic analysis is improved when it includes language beyond the spoken modality. This paper uses sign language data to explore and advance cross-linguistic typologies of *reflexives*, constructions expressing that co-arguments of a predicate are also co-referent. In doing so, we also demonstrate that the lexical item **KENDİ** in Turkish Sign Language (*henceforth*, TİD) can function as a traditional reflexive, in addition to its previously documented emphatic functions. We further show that **KENDİ** is a DP-type reflexive, which helps to explain the emphatic usages of **KENDİ** that have been the focus of previous research. We end by outlining a plan for future research that can further probe and unify the superficially distinct functions of **KENDİ** and the typology of anaphoricity across modalities. Data for the present research comes from recently conducted fieldwork interviews with two signers of the İstanbul dialect of TİD, both of whom have been exposed to TİD since birth.

## Introduction

Sensitivity to event participant structure is evident in the earliest stages of language acquisition (Pinker, 2013; Pace et al., 2016) and distinguishing the participants in an event is so fundamental to human language that it is present even in homesign (Goldin-Meadow et al., 2009). Interestingly, languages also universally have mechanisms for indicating that event participants are *not* distinct: reflexive constructions. Conceptually, reflexivity is a specific type of dependency relation between two arguments of a predicate where the two arguments are co-referent. Languages of the world have different strategies to mark this relation (Faltz, 2016/1977), as we discuss in more detail in “Background on Reflexivity.” In English, for example, this relation can be marked *via* reflexive pronouns (1).


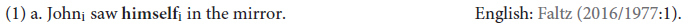


Compared to the research on spoken languages regarding reflexivity (Frajzyngier and Walker, 2000; Büring, 2005b; Geniusiene, 2011; Faltz, 2016/1977, i.a.), there is limited literature on sign languages. This is partly because the linguistic study of sign languages is a fairly new endeavor. Though scattered earlier documentation exists (e.g., Desloges, 1779), linguistic analysis of sign languages began in earnest with Stokoe’s (1960) work on American Sign Language (*henceforth*, ASL). Though Stokoe made some observations regarding ASL syntax, his focus on the phonetics and phonology of signs left “much more” to do “in establishing exactly what are the structural principles of the sign language sentence” (Stokoe, 1960:32). Though much progress has been made since then (as one sees from all the work on sign languages cited in this paper as well as the other contributions to this volume), Stokoe’s (1960) statement still holds: there is still a tremendous amount of research to be done.

The research that has been done has shown that sign languages employ several strategies to form reflexive structures. For example, Kimmelman (2009) reports that ASL^1^, Russian Sign Language, Dutch Sign Language, Israeli Sign Language, and Croatian Sign Language all have reflexive pronoun strategies. Because reflexivity is present in some form across various languages and language families (Frajzyngier and Walker, 2000; Büring, 2005b; Geniusiene, 2011; König et al., 2013; Faltz, 2016/1977, to name a few) and across modalities, it may be a universal phenomenon of language. In that case, describing and analyzing reflexivity in understudied languages like sign languages provides an opportunity to expand existing accounts and holds a great deal of importance in testing our existing generalizations about argument structure and reflexivity. This is the aim of the present paper. We hope to contribute to the ongoing endeavors to document, describe, and analyze sign languages in the pursuit of a better understanding of human language.

**FIGURE 1:**
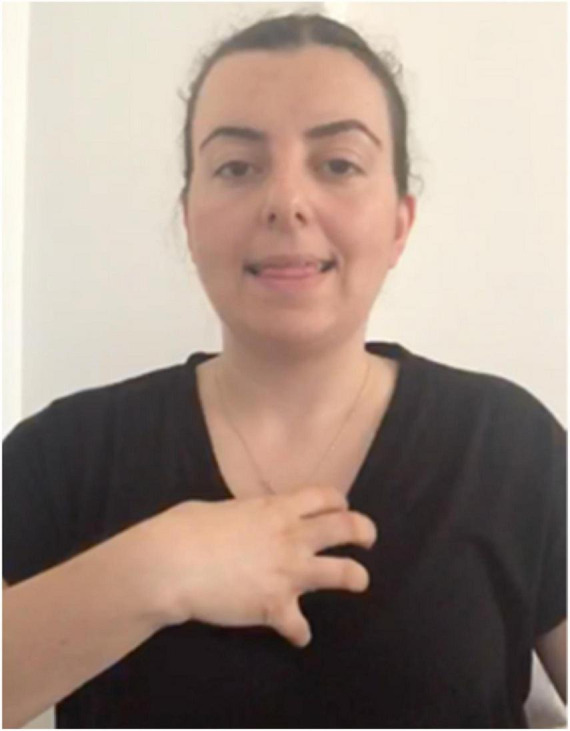
**KENDİ**.

Our focus will be the lexical item **KENDİ** in Turkish Sign Language (*henceforth*, TİD), which is produced by tapping in the middle of the chest with an open hand, fingers bent inward, as depicted in Figure 1.

There has been little work on **KENDİ** in the existing literature, much of it limited to in-passing observation that the sign **KENDİ** exists. Zeshan (2002) and Sevinç (2006:16) briefly note that the form is attested but observe only an emphatic function (2a). In their recent grammar of TİD, Kelepir (2020a), citing data from Dikyuva et al. (2017), nevertheless label **KENDİ** as a reflexive pronoun (2b):


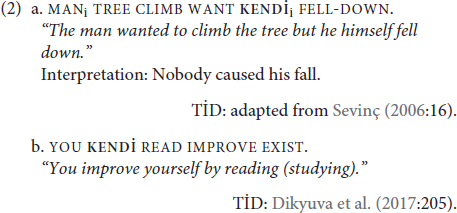


Our study looks to build upon these limited observations to examine if **KENDİ** can be used both as a traditional reflexive [as Dikyuva et al. (2017) and Kelepir (2020a) suggest] and/or as an emphatic marker [as Zeshan (2002) and Sevinç (2006) observed]. We focus on the İstanbul dialect of TİD. The layout of the paper is as follows: “Background on Reflexivity” summarizes the notion of reflexivity and provides an overview of the previous literature on reflexives in signed and spoken languages. “Methodology” explains our methodology of data collection. “**KENDİ** Marks Co-referential Relations” lays out the co-referential properties of **KENDİ** as a traditional reflexive. Having established that **KENDİ** can function as a traditional reflexive, we then turn to where **KENDİ** stands within reflexive typologies. Building on the data from earlier sections, “Co-referential Relations With **KENDİ**” further probes the syntactic and semantic properties of **KENDİ** in its traditional reflexive function. The functions of **KENDİ** beyond its traditional reflexive role is the focus of the final section before we close by summarizing our findings and laying out directions for future research.

## Background on Reflexivity

In this section, we will overview basic properties of reflexivity that are relevant for this study and summarize the reflexivity patterns that have been documented in spoken and signed modalities.

### Reflexivity in Spoken Languages

The literature on reflexivity suggests that it is a universal part of language, observed in many languages across different language families, albeit encoded with different grammatical mechanisms. With respect to the strategies that encode reflexivity, we can talk about two main kinds of reflexivity: *lexical* reflexivity and *grammatical* reflexivity. Lexical reflexivity, which is also sometimes called *inherent* reflexivity, is a phenomenon we observe on predicates that express events that are prototypically done to oneself, such as “bathe”. The default interpretation of an intransitive sentence like *I bathed* in English is reflexive, *I bathed [myself].* Predicates like “bathe” can, however, express non-reflexive events, as with the transitive variant *I bathed the dog* in English. Lexical reflexives in English are also often compatible with (redundant) grammatical reflexivity, as in *I bathed myself* (emphatic interpretations, which we discuss below, may be more natural here). Here, the reflexivity relation is not only expressed *via* the semantics of the verb but also the argument structure of the utterance. Grammatical reflexivity manifests itself either *via* marking on the verb (a) or marking on the arguments (b):


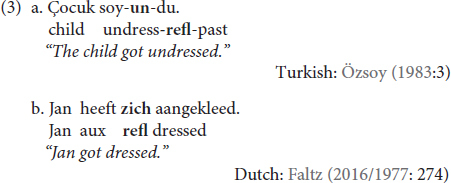


In (3a), the reflexive marker {-un}^2^ is a verbal suffix. As for (3b), Faltz (2016/1977) observes that the reflexive pronoun “zich” in (3b) links the object of the verb to the subject, marking that the do-er and the patient are the same referent.

There is also variation *within* the categories of verbal and argumental reflexivity marking. Verbal reflexivity includes affixes like *-un*- in Turkish (3a) and clitics, such as the French *se* in *s’habiller* (“to dress oneself”). As for argument marking, we observe both free standing reflexive pronouns such as *zich* in (3b) as well as bound morphemes that shift a stem to a reflexive meaning (e.g., -*self* in English). It is not uncommon for languages to exhibit multiple reflexive strategies, or to combine them as part of a complex reflexive construction.

What unites these different strategies is that they are all subject to certain structural constraints. First of all, a reflexive requires an antecedent for co-reference, and the relationship between them is often called *binding*. Argument reflexives require being bound by a potential co-referent (4a-b) and are usually restricted as to what can bind them within what structural domain and/or configuration (4c-d), e.g., intra- or inter-clausal (see, among others, Chomsky, 1981; Reinhart and Reuland, 1993; Faltz, 2016/1977).


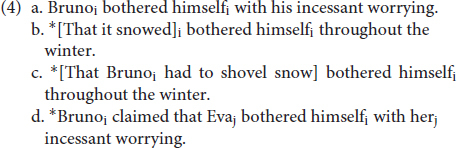


The literature on reflexives has long attempted to unify the structural constraints on the reflexivity. However, pinpointing these constraints isn’t always an easy task and is further complicated by the fact that reflexives are commonly observed to serve other functions (Reinhart and Reuland, 1993; Faltz, 2016/1977; Déchaine and Wiltschko, 2017 i.a.) that may not be subject to the same structural constraints as a traditional reflexive. Emphatic anaphors (5a) refer to one of the participants within the event, but their function is to put focus on or set apart a particular participant without affecting the argument structure of the verb. Logophors (5b), on the other hand, are anaphoric items that can get their co-reference from outside of the structural domain of a traditional reflexive. Thus, emphatic markers (5a) and logophors (5b) still express co-reference, which makes them *anaphoric*. However, they do not necessarily express co-reference between arguments of the predicate, so they are not *reflexive*.


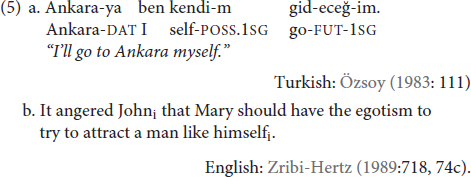


Such multi-functionality has already been observed for **KENDİ** (Zeshan, 2002; Sevinç, 2006; Dikyuva et al., 2017), which we discuss in more detail below. However, what we first aim to show in this paper is that **KENDİ** can be used as a traditional reflexive. Before we move on to discussing that, however, we first provide a review of the existing reflexivity literature on *sign* languages.

### Reflexivity in Sign Languages

As in other domains of linguistic structure, research on reflexivity in sign language is limited. However, the research that has been done observes key similarities across modalities. Sign languages, too, employ two main strategies to mark reflexivity: on the verb (6a), or on the argument of the verb (6b):


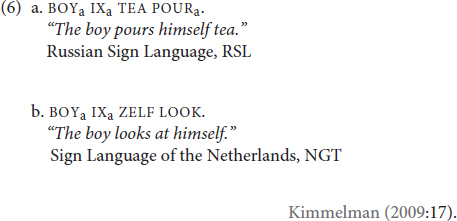


The verbal reflexivity of (6a) is expressed *via* the spatial agreement markers, indicated by the subscripted ‘‘a’’^3^. Here, the direction of the object agreeing verb’s path movement agrees with the subject, linking the grammatical object and the subject of the sentence in co-reference. Kimmelman (2009) also reports argumental reflexive pronouns in NGT, glossed as ZELF in (6b), similar to English *-self*. This parallelism across modalities is not unexpected considering that reflexivity reflects event participant structure, which may be conceptually and linguistically fundamental. This parallelism aside, what sign languages bring to the table is the way they use space for reference, which may lead to modality-specific effects on reflexivity. The role of space in modulating reflexivity is evident in (6a). These reflexivization strategies are not specific to the exemplified languages. Table 1 below shows various reflexivization strategies attested in sign languages.

**TABLE 1:** Reflexivization Strategies Attested in Sign Languages.

	Verbal marking
	-Israeli Sign Language (ISL, Meir, 1998) -American Sign Language (ASL, Sandler and Lillo-Martin, 2006 i.a.) -Sign Language of the Netherlands (NGT, Kimmelman, 2008) -Italian Sign Language (LIS, Branchini, 2020) -German Sign Language (DGS, Loos, 2020)

	**Argumental marking**

Free form reflexive pronouns	-American Sign Language (ASL, Liddell, 1980; Lillo-Martin, 1995; Mathur, 1996; Wilbur, 1996; Sandler and Lillo-Martin, 2006; Koulidobrova, 2009, 2011; Fischer and Johnson, 2012; Wilkinson, 2013; i.a.) -Israeli Sign Language (ISL, Meir, 1998) -Croatian Sign Language (CSL, Cicilianić and Wilbur, 2006) -Turkish Sign Language (TİD, Zeshan, 2002; Sevinç, 2006; Dikyuva et al., 2017; Kelepir, 2020a) -German Sign Language (DGS, Mehling, 2010) -Sign Language of the Netherlands (NGT, Kimmelman, 2008) -Russian Sign Language (RSL, Kimmelman, 2008) -Catalan Sign Language (LSC, Navarrete-González et al., 2020) -Italian Sign Language (LIS, Mantovan, 2020)
Derived reflexive pronouns	-Italian Sign Language (LIS, Mantovan, 2020)
Personal pronouns	-Israeli Sign Language (ISL, Meir, 1998) -Croatian Sign Language (CSL, Cicilianić and Wilbur, 2006) -Russian Sign Language (RSL, Kimmelman, 2008)

The multi-functionality of reflexive markers is also relatively well-documented in sign languages. As an example, SELF in ASL can also functions as copula (7a) and as an emphatic marker (7b):


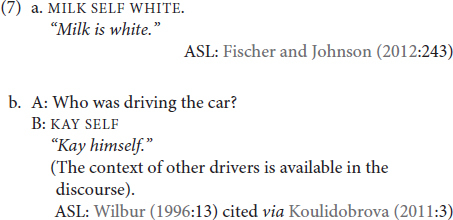


All in all, the existing work, though limited, shows that reflexivity in sign languages is compatible with certain aspects of existing typologies. However, it is also the case that sign languages can provide unique and novel data to further explore the phenomenon of reflexivity, including how usage of space affects referential relations (Schlenker, 2018). Before turning to what TİD shows us about reflexivity, we first briefly explain how we collected and analyzed data in this study.

## Methodology

The data for this work comes from fieldwork sessions with two Deaf adult female signers of the İstanbul variety of TİD. Both have been exposed to TİD since birth. The sessions took place 2020–2021 and were conducted online *via* Facetime and Zoom due to the limitations on travel and in-person meetings during the COVID-19 pandemic. The language of interaction was TİD, though both consultants also have proficiency in written Turkish. We have utilized acceptability judgments and having the consultant describe contexts and situations using **KENDİ** as methods of data elicitation. Only one consultant at a time was present for each fieldwork session. Because of quality issues that can arise in videoconferencing, the data reported here were also recorded separately by one of the consultants and can be accessed in an online repository (file names correspond to example numbers): https://tinyURL.com/KendiRepository.

## KENDİ Marks Co-Referential Relations

In the previous section, we described the basic patterns of where and how co-referentiality and, specifically, reflexivity is marked in language. In this section, we will look further into co-referential dependencies in TİD, and the core properties of co-referentiality marked by **KENDİ**. Our aim here is to lay the groundwork for the more detailed discussion of the distribution of **KENDİ** in later sections.

As noted above, co-reference is structurally constrained and certain classes of DPs are restricted in the co-reference relations they can enter into. Büring (2005b) categorizes the possible co-referential relations between DPs as obligatory co-reference (8a), obligatory non-co-reference (8b), and optional co-reference, i.e., ambiguity (8c):


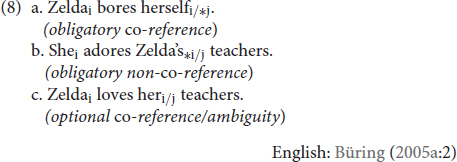


In (8a), the reflexive *herself* has to refer to Zelda, as indicated by the subscripted referential indices. In (8b), the pronoun *she* has to refer to an entity *other* than Zelda. Lastly, in (8c), the possessive pronoun *her* allows reference to either Zelda or an individual outside of the clause, a discourse referent. The Binding Theory (Chomsky, 1981) aims to account for these patterns of obligatory co-reference (Principle A), obligatory non-co-reference (Principle C), and optional co-reference (Principle B):


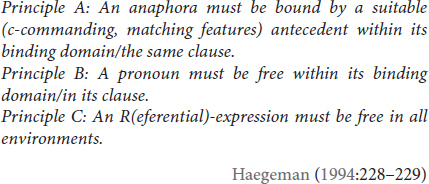


In (9), we document these patterns in TİD:


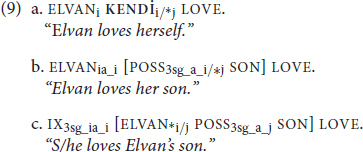


**KENDİ** in (9a) must refer to the subject, ELVAN, as indicated by the subscripted referential indices. This is obligatory co-reference, as one would expect of a traditional reflexive governed by Principle A. Skipping ahead to (9c), we again observe a familiar pattern: the sign name ELVAN and the 3rd singular subject cannot be co-referent. This is obligatory-non-co-reference, governed by Principle C. The structure that gives rise to optional co-reference ambiguity in (8c), however, patterns differently in TİD (9b) due to the spatialization of the possessive marker (see Cormier et al., 2013 for a discussion of pronominal spatialization in sign languages). POSS in TİD, as in many other sign languages, spatially indicates its referent (here, ELVAN). Thus, we have obligatory co-reference with a *non*-reflexive pronominal not so much because binding works differently in TİD (9b), but because spatialization can prevent certain ambiguities from arising in the sign modality (Quer and Steinbach, 2015).

Interestingly, optional co-reference *is* possible if the overt possessive is removed entirely, as in (10).


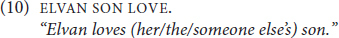


The ambiguity here is highly dependent on context and includes interpretation as definite nominal (“the son”). Ambiguity on a par with (8c), however, can arise due to the use of a null possessive. Because it is null, the possessive is not spatialized as in (9c). The null possessive can be co-referent with the overt (subject) argument (ELVAN) by default/without special context (on a par with the preferentially bound interpretations observed elsewhere, cf. Reinhart, 1983; Kehler and Büring, 2007). However, it can also refer to another contextually salient referent, as in (11):


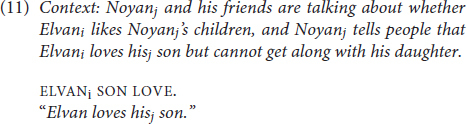


Turning next to the structural distribution of these three co-referential patterns, Büring (2005b) shows that lack of an antecedent in a mono-clausal setting affects each type of DP differently (12):


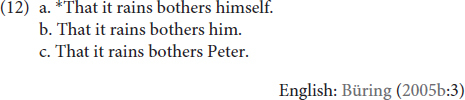


There are also structural constraints on where an antecedent can be when it is present, and this is where notions of reflexivity are key. The sentences in (13) express a reflexive event. Unsurprisingly, that reflexive event can be described using an obligatorily co-referent reflexive pronoun (13a). The reflexive event cannot be described using an obligatorily non-co-referent R-expression (13c), nor can it be described using a non-reflexive pronoun (13b).


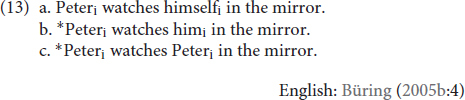


Parallel structural constraints hold in TİD. Like (12a), (14a) is ungrammatical because **KENDİ** requires a morphosyntactic antecedent (14a), unlike a non-reflexive pronoun such as IX_3sg_ in (14b) or an R-expression such as ELVAN in (14c). Moreover, as in (13), we see that only **KENDİ** can be used in a reflexive environment like (15).


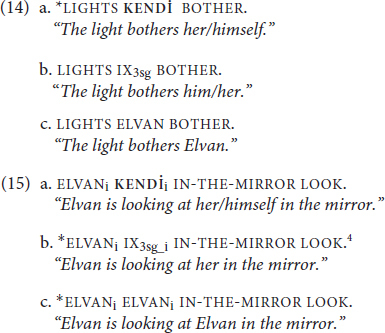


Thus, **KENDİ** appears to behave like a traditional reflexive that (i) requires a co-referential antecedent that is (ii) in the right structural configuration.

These morphosyntactic constraints pertain to what is meant by “binding domain” in Principles A-C. Binding domains appear to be sensitive to structural proximity, often called locality, which we illustrate here with clause boundedness. The sentences in (16) present the three types of DPs in the object position of a subordinated clause in English, with their potential antecedent in the subject of the matrix clause.


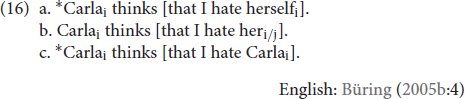


The boundary between the matrix clause and the subordinate clause seems to also function to demarcate binding domains. Thus, the antecedent for the reflexive in (16a), as compared to (8a) or (13a), is “too far away” to satisfy its binding requirements. In the case of a non-reflexive pronoun, the added distance of (16b) relative to (13b) allows the optional co-reference to emerge. Lastly, (16c) affirms that co-referential dependency, even when not local, between an R-expression and a potential antecedent is ungrammatical. Recall that this dependency was ungrammatical when it was local in (13c), too. Focusing on the comparison of (13a) and (16a), these data show that a traditional reflexive *requires* binding by an antecedent within its own *local* domain. This then would predict that if **KENDİ** is indeed a traditional reflexive, we should see evidence of structural sensitivity and locality constraints (though they may not match, exactly, those of English). The TİD equivalents of (16) are presented in (17), and the data in (17a) show that this prediction is borne out. (17a) is only grammatical when **KENDİ** is bound by an antecedent in its local domain (IX_1sg_j_) like in (16a), as opposed to an antecedent outside of it (ELVANi). As for the pronoun in (17b), we again observe the sentence is rendered grammatical as long as the pronoun is bound by a co-referent outside of its local domain, as opposed to being locally bound. Moreover, we see that an R-expression in TİD (17c) is degraded if it has an antecedent *at all*, even if that antecedent is in a different domain (though in TİD the judgment is that the sentence is highly marked, not fully ungrammatical):


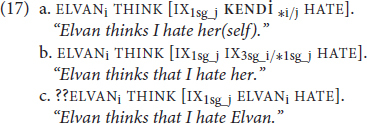


Thus, in many respects, TİD aligns with the principles of binding theory. However, these principles are under debate, and a common critique is that there is not strict complementary in the distribution of the three types of DPs. Our key observation here, though, is that **KENDİ** is only grammatical when bound by an antecedent that is syntactically proximal to it, such as (IX_1sg_j_) in (17a), as opposed to an antecedent outside of it (ELVAN_i_). The basics of co-referential relations among nominals in TİD tells us that **KENDİ** behaves like a traditional reflexive. However, reflexives aren’t a homogenous class, and reflexives with different morphosyntactic and semantic encodings may be subject to different restrictions (Thráinsson, 1976; Hellan, 1988; Reuland, 2011; Déchaine and Wiltschko, 2017). In the next section, we explore what type of traditional reflexive **KENDİ**is, using the typology proposed by Déchaine and Wiltschko (2017). As will become clear in below, this typologically informed analysis of **KENDİ** can help us better understand previous claims in the literature.

## Patterns With KENDİ Relative to Typologies and Analyses of Reflexivity

In the previous section, we summarized the basic co-referential relations in TİD and showed that **KENDİ** can function like a traditional reflexive. As we noted briefly above, however, reflexives are not a uniform class in many respects, including their syntactic category. Thus, we next ask what *type* of traditional reflexive **KENDİ** is, using the syntactic typology of reflexives proposed by Déchaine and Wiltschko (2017). Déchaine and Wiltschko (2017) divide reflexives into five types, and provide examples of languages with each type of reflexive (Table 2).

**TABLE 2:** Déchaine and Wiltschko’s (2017) typology of reflexives.

Language	Example	Category	Syntactic parallel	Other functions
French	se	Clitic	Case	Reciprocal, middle, inchoative, applicative
Halkomelem	Bound noun	Bound noun	Inalienable Possession	N-compound, numeral classifier, applicative
Shona	zvi̇-	Agreement Marker	Classifier	Agreement, evaluative, adverb
Cree	-iso	Intransivizer	Valency	Medio-reflexive, inchoative
English	xself	DP	Possessor	Logophor, emphatic pronoun

The main types of reflexives are: clitics, bound nouns, agreement markers, intransitivizers, and DP constituents. In addition to differing in syntactic category, the different types also exhibit slightly different structural patterns and contribute somewhat different semantics (despite all being a reflexive).

One such difference is that the multi-functionality of the reflexive marker differs depending on its type. The typology laid out by Déchaine and Wiltschko (2017) can be used to determine where **KENDİ** stands among reflexive markers, laying more solid groundwork for further analysis of **KENDİ** and co-reference relations in TİD. We begin by using Déchaine and Wiltschko’s (2017) diagnostics to identify the morphosyntactic category of the reflexive **KENDİ**. The results of these diagnostics show that **KENDİ** behaves like a DP-type reflexive. The classification of **KENDİ** as a DP-type reflexive leads to predictions about its behavior and functions beyond marking traditional reflexivity, which we discuss further in “The Function of **KENDİ** Beyond Traditional Reflexivity.”

### KENDİ Is Not a Clitic

Much like traditional reflexives are semantically dependent on an antecedent, clitics are morphologically and phonologically dependent on a host. They cannot bear stress, and often come in a reduced phonological form, as exemplified by the reflexive clitic *m’* in (18), which is a reduced form of the pronominal *me*. As the gloss suggests, the reflexive clitic *m’* forms a phonological unit with its verbal host (*auto-suggère)*. Moreover, though French is typically a postverbal object language and *m’* is expressing the reflexive object of the predicate, the clitic appears in a preverbal position. Thus, the clitic has characteristic properties in terms of its morphophonology and its morphosyntactic distribution.


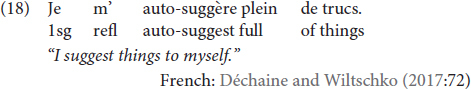


Turning to TİD, a default SOV language, note first that the typical position of object **KENDİ** is in the standard pre-verbal position, as illustrated in (9a) and similar examples. This distributional pattern is not inherently at odds with a clitic analysis, but if **KENDİ** were a clitic, we would expect it to form a phonological unit with a preceding or following element. However, as illustrated in (19), **KENDİ** can be morphophonologically separated from the verb (its following element) by an intervening adjunct (as illustrated in the repository video, the adjunct intervening in (19a) continues throughout the production of the verb, while the adjunct in (19b) is clearly sequential):


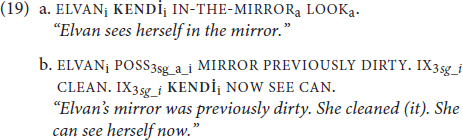


**KENDİ** is also morphophonologically independent from the elements that *precede* it. For example, **KENDİ** is adjacent to its antecedent, ELVAN in (19a), but separated from ELVAN by the intervening adverb NOW in (20).


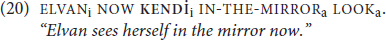


Another piece of evidence that **KENDİ** is not a clitic is that it can dislocate to the left periphery, giving rise to a contrastive topic meaning (21).


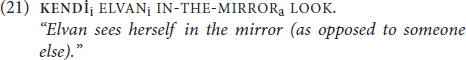


In TİD, contrastive items are often observed with an eyebrow raise that is articulated simultaneously with the contrast-associated item (Gökgöz and Keleş, 2020 section 4.2). It is also the case that non-manual spread (annotated with the line above the manual sign glosses) (21) in sign languages has been associated with marking phrasal boundaries (Pfau, 2005, 2006). The non-manual marker associated with focus in (21) is not spreading over the subject ELVAN. Therefore, (21) not only shows that **KENDİ** can be linearly dislocated from what would have been its most plausible host, but also shows that it forms its own prosodic unit. This, then, backs up the narrative that **KENDİ** is a morphophonologically independent form.

**FIGURE 2:**
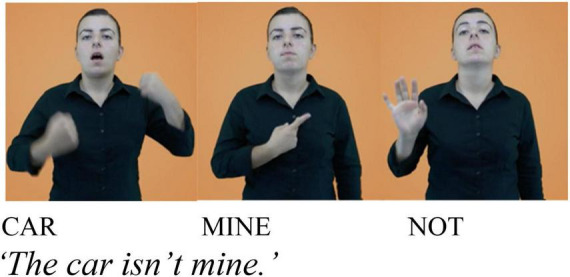
Adapted from Makaroğlu and Dikyuva (2017, entry: “değil”). Open-source image available from the online TİD dictionary: http://tidsozluk.net.

**FIGURE 3:**
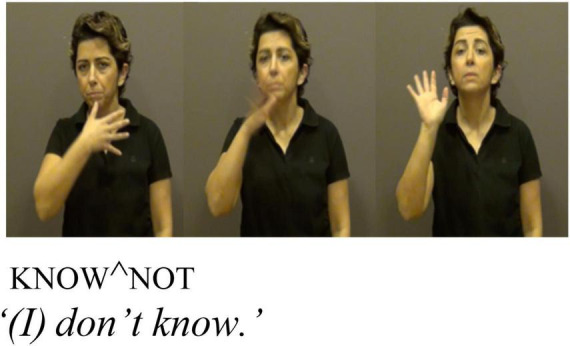
Adapted with permission from Kelepir (2020d: P4, 3.5.1.1).

Comparing **KENDİ** to other clitics that *have* been documented in TİD -namely, the clitic form of negation—also reveals differences. Zeshan (2003, 2004) and Kelepir (2020d:3.5.1.1) observe that manual negation in TİD can occur as a free form (Figure 2) or a clitic (Figure 3)^5^. In its cliticized form, negation loses its syllabicity (reduced movement, shorter duration) and assimilates to the location of its host, instead of the neutral signing position used in Figure 2.

**FIGURE 4:**
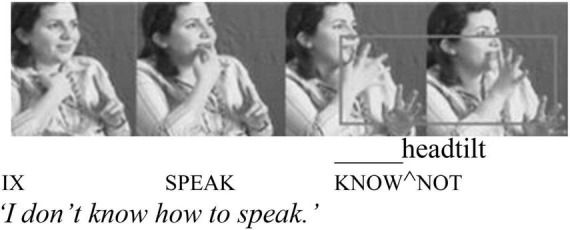
Adapted with permission from Gökgöz (2009:20).

**FIGURE 5:**
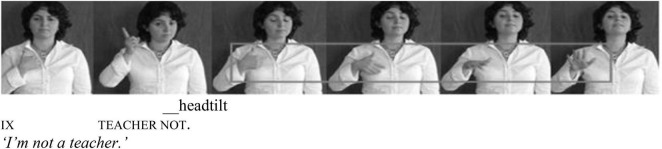
Adapted with permission from Gökgöz (2009:21).

Unlike clitic negation, the signing position of **KENDİ** does not get assimilated to that of its host. This could be due to the body-anchored nature of **KENDİ**. However, there are other ways **KENDİ** differs from the negative clitic. Building on Zeshan and Kelepir’s analyses, Gökgöz (2009) found that the non-manual marker for negation, a head tilt, patterns differently with the clitic and non-clitic form. With cliticized negation, the head tilt associated with negation spreads onto its morphophonological host, as indicated by the line above KNOW and cliticized ^NOT in Figure 4. With non-cliticized, free negation, however, the non-manual marker only spreads over the negation itself (Figure 5).

Here, too, (21) provides the relevant evidence: we might expect the eyebrow raise in (21) to spread onto ELVAN too, had **KENDİ** cliticized to it. Thus, based on evidence from intervening items and the properties of clitics in TİD, the relationship between **KENDİ** and preceding or following elements is linear adjacency, not morphophonological dependency, as would be expected of a clitic.

### KENDİ Is Not a Bound Noun

Bound noun reflexives are attested in languages such as in Halkomelem, where body part nouns attached to the predicate can be interpreted as co-referential with an argument, either the subject in an intransitive verb form (22a) or the object in a transitive verb form (22b). In the case of the predicate with an intransitive marker (-*em*) in (22a), *only* the reflexive interpretation is possible.


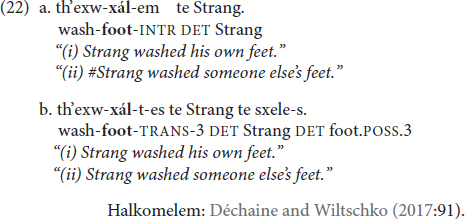


Data like that presented in the previous section also argue against a bound noun analysis of **KENDİ**’s. **KENDİ** does not display the morphophonological characteristics of a bound element.

### KENDİ Is Not an Agreement Marker

Our next step is to check if **KENDİ** is a verbal agreement marker. As discussed earlier, marking reflexivity on the verb is a commonly employed phenomenon in signed (and spoken) languages. Moreover, sign languages often make use of space for modulating agreement, and we know that TİD is a sign language that marks agreement spatially (Gökgöz et al., 2020 section 2.1.2.3.1). Spatial markers on predicates parallel agreement markers in tracking the event participants [see contributions to Lillo-Martin and Meier (2011) for discussion]. In fact, even intransitive predicates with a single argument can spatialize this way (Costello, 2016). However, we argue that this is not what **KENDİ** is doing for two reasons: (i) it marks reflexivity without being assigned a locus and (ii) still allows the verbal reflexive agreement marker to appear (if compatible with the predicate in general; Kelepir, 2020b section 3.1).

Before elaborating on those arguments, we first provide an example of a reflexive that is of the agreement marker type in Shona. The reflexive *-zví* in (23) has the distribution of an object agreement marker in the morphological template of Shona^6^ (note that Shona also has a non-reflexive *-zvì* that differs in tone from the high-toned reflexive *-zví*):


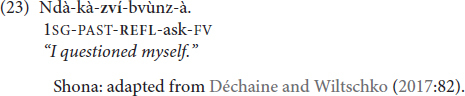


As discussed above, **KENDİ**, as compared to *-zví*, does not form a morphophonological unit with the verb. Indeed, **KENDİ** is not even obligatorily adjacent to the verb, as one might expect of an agreement marker in general. Moreover, **KENDİ** is body-anchored and does not make use of an assigned locus in the signing space. Thus, it’s quite unlike how agreement *is* marked in TİD (Gökgöz et al., 2020:2.1.2.3.1) and in other sign languages (Cormier, 2012:124–125; Sandler, 2012: 44–45). Finally, if **KENDİ** *were* a kind of less common agreement marker in language that does *not* form a unit with the predicate it’s marking “on”, and an almost unattested kind of agreement marker in sign language that does not make use of space, we would expect it to show up post-verbally, because that’s where functional items typically occur in TİD (Gökgöz, 2020).^7^ The default position of **KENDİ**, however, is preverbal. Therefore, as above, both cross-linguistic and language-internal patterns argue against this analysis of **KENDİ**.

### KENDİ Is Not an Intransitivizer

As for the intransitivizer category, Déchaine and Wiltschko (2017) point to the *-iso* suffix in Cree as an example. They observe that patient/object marking is absent on the verb when the reflexive marker is present (24a), but present then the verb is used non-reflexively (24b):


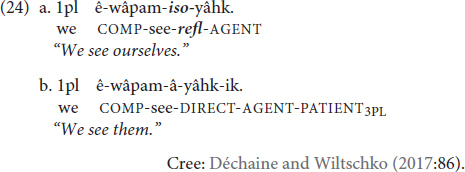


**FIGURE 6:**
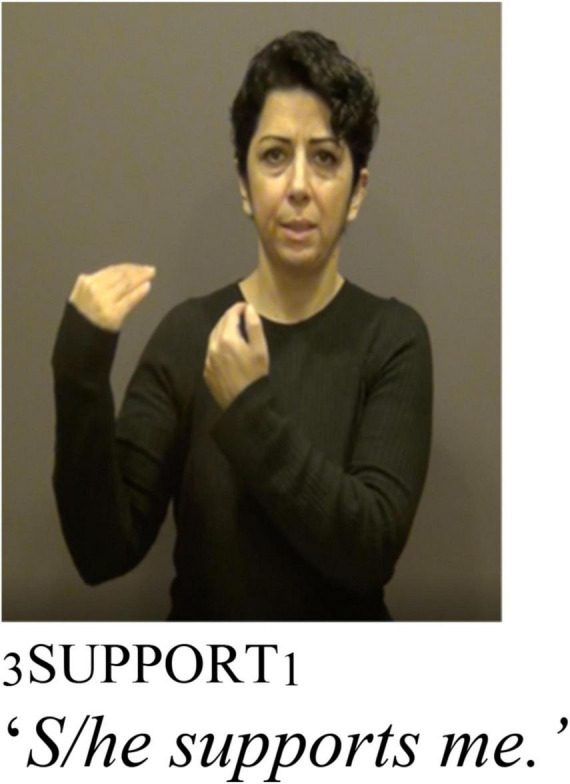
Adapted with permission from Kelepir (2020c: P4, 3.1.1.2).

**FIGURE 7:**
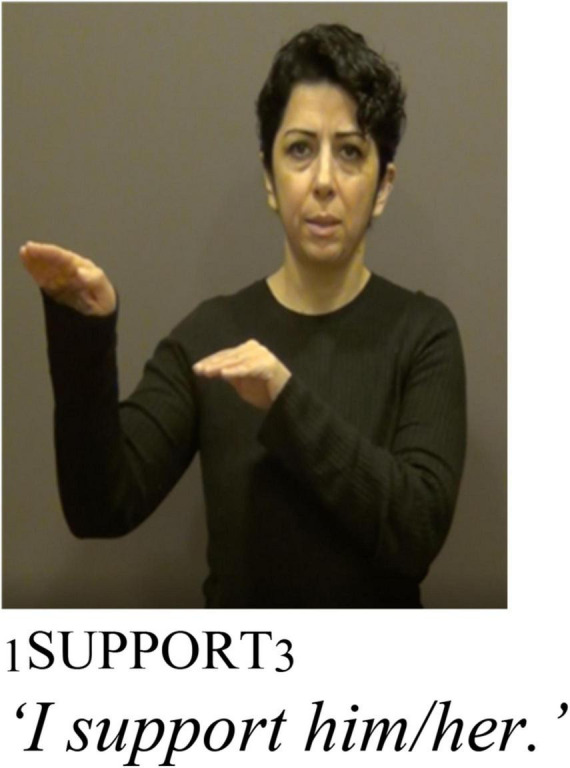
Adapted with permission from Kelepir (2020c: P4, 3.1.1.2).

As described above, TİD and other sign languages can incorporate spatial locations to mark agreement. TİD also displays object agreement on some verbs through palm orientation (Kelepir, 2020c section 3.1.1.2). Figures 6, 7 illustrate how palm orientation marks object agreement in TİD. In Figure 6, the object is 1st person and the palm orientation is toward the signer, while in Figure 7 the palm orientation is toward an established spatial locus away from the signer’s body, the orientation of a 3rd person marker.

This palm orientation agreement marker is present in non-reflexive predicates like PROTECT or SUPPORT. Crucially, however, it is also present in reflexive usages of the same predicates. This is illustrated for reflexive and non-reflexive usages of the predicates PROTECT and SUPPORT in (25). In a sentence like (25a), the 3rd person palm orientation is toward a spatial locus away from the signers’ body, whereas in (25b) there is the reflexive marker **KENDİ** *as well as* palm orientation toward the signer’s body, just like Figure 6.^8^ Note that the signer has omitted the subject agreement marker in (25b), as has been observed elsewhere in sign languages (Padden, 1988, 1983).


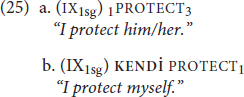


Thus, **KENDİ** does not manipulate the argument structure like the intransitivizer *-iso* in Cree does, and we can conclude that **KENDİ** is not an intransitivizer because it co-occurs with a marker of transitivity, the palm orientation marker of object agreement.^9^

### KENDİ Is a DP Reflexive

So far, we have seen that **KENDİ** does not align with the patterns of a clitic, bound noun, intransitivizer, or agreement type of reflexive. However, there is another kind of reflexive in the typology: a DP reflexive.


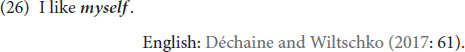


DP reflexives are basically reflexives that act like any DP, except for the specific dependency relation that they require a co-referring antecedent. Previous examples have shown that **KENDİ** is a reflexive that behaves like any DP (object) argument of the verb: it occupies an A position as the canonical object in the sentence and, moreover, can be dislocated to an A′ position the left periphery.

Recall that Déchaine and Wiltschko’s (2017) typology includes syntactically parallel items for each of the reflexive types. For DP type reflexives, the parallel they observe is possessors. Note that in some DP type reflexives, the connection with possessors is transparent; the reflexive form in English contains a possessive: *my*self. For **KENDİ**, the connection with possessives is twofold. One, **KENDİ** can be combined with an overt possessor like the English “*xself*”, as in (27).


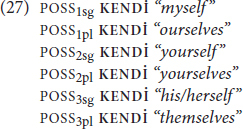


Two, **KENDİ** itself can also be used as an independent possessive (28), though this usage isn’t very common.^10^


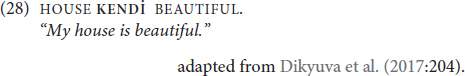


There are also cases of complementary distribution, where possessive usages of **KENDİ** block another possessive:


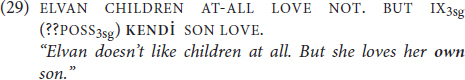


With respect to reflexive multi-functionality, additional functions of the DP type reflexives that Déchaine and Wiltschko (2017) observe are serving as emphatic anaphors (30a) and a logophors (30b):


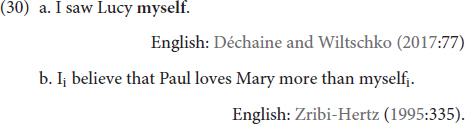


As a reminder, emphatic anaphors are used to focus some aspect of the event or the referent’s role in the event, often from a non-argument position, and logophors are anaphoric elements that seemingly skirt binding principles by getting their co-referent from a discourse antecedent outside of their local domain. Thus, emphatic anaphors and logophors are reflexive *forms* that are in grammatical positions that are not associated with reflexivity (emphatic anaphor), or have non-prototypical antecedents (logophor). We are currently investigating whether a logophoric usage of **KENDİ** possible, but we already know from previous observations by Zeshan (2002) and Sevinç (2006) that **KENDİ** does indeed function as an emphatic anaphor. We elaborate on the emphatic anaphor usage of **KENDİ** in “The Function of **KENDİ** Beyond Traditional Reflexivity.”

To summarize, our fieldwork reveals that **KENDİ** in TİD can function as a traditional reflexive, and its previously observed usage as an emphatic anaphor is connected to its status as a DP-type reflexive, similar to English *xself*, and unlike other syntactic categories of reflexives discussed above. Moreover, we have seen that **KENDİ** shares other features that characterize DP-type reflexives, such as a structural parallelism with possessors. In the next section, we further explore the traditional reflexive usage of **KENDİ**, providing a more detailed description of its binding domain and its antecedents.

## Co-Referential Relations With KENDİ

We have thus far provided some basic observations regarding co-referential relations in TİD and shown that **KENDİ**exhibits behaviors consistent with a traditional reflexive. We have also argued that **KENDİ** is a DP-type reflexive. In this section, we will explore **KENDİ** as a traditional reflexive in more detail and discuss its relation to potential antecedents in local and long-distance binding domains. We first examine whether **KENDİ** can be bound by null antecedents as well as overt ones, and then the clausal location of these antecedents.

### Antecedents

A defining characteristic of a traditional reflexive is that it requires an antecedent. Whether this antecedent must be overt or not depends on whether the language in question allows null arguments. This is illustrated for **KENDİ** by the contrast in (31), where (31a) contains an overt antecedent (ELVAN) for **KENDİ** and is grammatical, but (31b) lacks an overt antecedent and is ungrammatical.


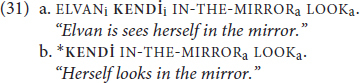


Judgments like those for (31a) and (31b) are in “out of the blue” contexts. However, language is rarely used in truly out of the blue contexts. Given that TİD permits null arguments, we would predict that **KENDİ** can be licensed by covert antecedents. The data in (32)-(33) illustrate that this prediction is borne out (“*e”* glosses the position of the null argument):


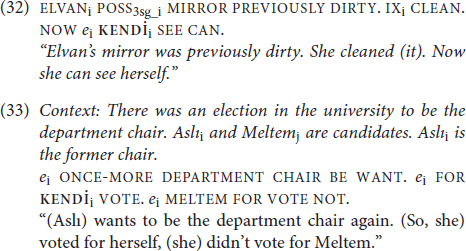


These data show us that the required antecedent for **KENDİ** can be null arguments that are licensed by earlier portions of the discourse (32) or by contextual salience (33). Like **KENDİ**, the null argument that binds the anaphor, *e_i_*, is subject to its own licensing conditions (*see* Kayabaşı et al., 2020 for a detailed discussion of null arguments in TİD).

These data affirm that **KENDİ** patterns like a traditional reflexive in requiring an antecedent, though independent patterns of null argument licensing in the language mean that this antecedent need not be overt. These findings are in line with existing research on null arguments and reflexive pronouns in other sign languages (Lillo-Martin, 1986; Bahan et al., 2000; Koulidobrova, 2012; Kimmelman, 2018; Kayabaşı et al., 2020 i.a.).

### The Structural Relationship Between KENDİ and Its Antecedent

We have already briefly described **KENDİ**’s relationship to overt and covert antecedents. In this section, we will talk about the structural logistics of **KENDİ** and discuss suitable structural positions for an antecedent to bind **KENDİ**, both in terms of hierarchy and proximity.

**FIGURE 8:**
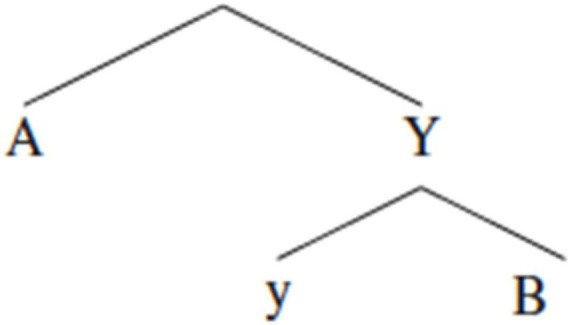
Representation of C-command between A and B.

For a traditional reflexive like **KENDİ**, Principle A (Chomsky, 1981) is usually interpreted as requiring c-command of the reflexive by its antecedent. C-command is a structural relationship between two nodes, X and Y, neither of which dominates the other, but where every branching node that dominates X, also dominates Y (Reinhart, 1976; Büring, 2005b, i.a.). Figure 8 illustrates how A can bind B but not the other way around since A c-commands B but not vice versa.

Crucially, there are patterns in language, like reflexivity, that show sensitivity to structural relationships like c-command. This is illustrated by the relationship between *Charlotta* and *her/herself* in (34). Note that the examples are structurally identical and *Charlotta* precedes the intended co-referential DP in both. Because *Charlotta* is “buried inside” the subject DP, however, it does not *c-command* the *her/herself* in either example. The resulting grammaticality differs depending on the use of a non-reflexive vs. reflexive pronoun. The non-reflexive pronoun in (34a) is grammatical because such pronouns are only optionally co-referent and do not need to be bound. However, a traditional reflexive is obligatorily co-referent, so the *herself* in (34b) *does* need to be bound. Consequently, expressing the co-reference *via* a reflexive, as in (34b), is ungrammatical.


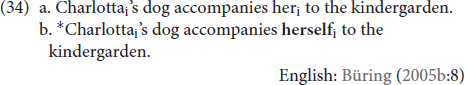


A similar example for TİD is presented in (35). Here, there are two possible antecedents for **KENDİ**, the possessor ELVAN_i_ and the full possessive DP [ELVAN_i_ POSS_3sg_i_ SISTER]_j_. Only [ELVAN_i_ POSS_3sg_ SISTER]_j_, however, is in a c-commanding relationship with **KENDİ**. As predicted, this is the only DP that can bind **KENDİ**:


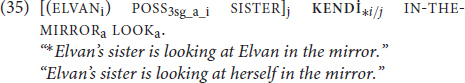


The fact that **ELVAN**_i_ cannot bind **KENDİ** tells us that TİD is no exception to the rule that the relation between the antecedent and a traditional reflexive is structurally determined, and one that cannot be characterized by linear precedence. Though critiques have raised questions about whether c-command accurately characterizes the structural constraints imposed on binding, what is relevant here is that (35) shows that there is a *structural* constraint at play. So far, we described what type of antecedents **KENDİ** can take and showed that it can be bound by both overt and null antecedents. Moreover, we have shown that the relation between **KENDİ** and its antecedent is subject to some type of structural constraint. To assess the proximity aspect of the antecedent-reflexive relation, we turn next to the pattern of **KENDİ** in subordinate sentences. In (36), **KENDİ** is in the object position of the subordinated predicate SEE, and co-reference with the subject (ELVAN) of the matrix predicate WANT is possible (either *via* direct binding or mediated by an intervening null argument, itself co-referent with ELVAN):


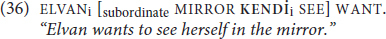


Note, however, there are no other viable antecedents in (36). In (37), we see how **KENDİ** behaves when multiple possible antecedents *are* present in the sentence.


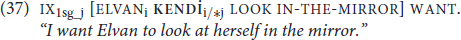


Here, too, **KENDİ** is in the subordinate object position, but now two overt and distinct referents are available to serve as antecedents: the matrix predicate subject, IX_1sg_j_, and the subordinate subject, ELVAN_i_. The only permitted antecedent for **KENDİ** in (37) is the closer subordinate subject. Note that the WANT type verbs in (36–37) are usually associated with non-finite sentential complements. In (38), we see the same binding patterns with THINK, a verb type that is often associated with finite sentential complements. There is currently very little known about finiteness in sign languages (and almost nothing known about this phenomenon in TİD) within the existing literature, but the patterns are the same across these predicate types.


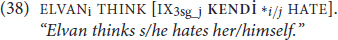


Together, (35)-(38) show us that **KENDİ** is sensitive to familiar, structural binding constraints of hierarchy and proximity. Having elaborated a bit on the co-referential relations of **KENDİ** as a traditional reflexive, we will now turn to **KENDİ**’s function beyond traditional reflexivity, emphatic anaphoricity.

## The Function of KENDİ Beyond Traditional Reflexivity

**KENDİ** has already been observed to be used as an emphatic anaphor (*see* Zeshan, 2002; Sevinç, 2006), as is commonly true of other DP-type traditional reflexives (Déchaine and Wiltschko, 2017). In this section, we will further explore the emphatic anaphor function of **KENDİ**.

### Two Types of Emphatics

Emphatic markers are anaphors that co-refer to a participant of the given event to cast focus on it and to contrast it from other participants in a possible set of participants (Kemmer, 1995; Stern, 2004, i.a.). They usually occupy non-argument positions, and they do not express reflexivity. Focusing on English, Ahn (2010) notes two distinct usages of the emphatic anaphor *xself* (here, *himself*), exemplified in (39). In (39a), *himself* creates argument focus on its event participant antecedent, a ‘‘specifically John and not someone else’’ meaning. In (39b), however, *himself* modifies the event (not an argument), and emphasizes that John performed the given event without help or the contribution of another causer/agent.^11^


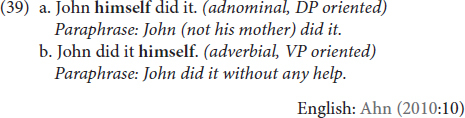


He labels the interpretation that arises from modifying the DP (39a) as “adnominal”, and the one that arises from modifying the DP as “adverbial” (39b).

In (40) we see the same two-way distinction with the emphatic function of **KENDİ**:


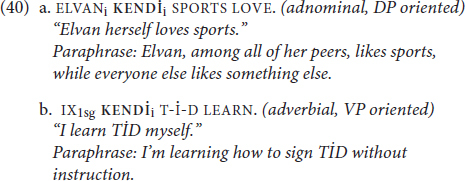


As the translations show, (39a-40a) and (39b-40b) resemble one another with respect to the emphatic contribution of *himself* and **KENDİ**.

Notice, though, that the different interpretations of *himself* in (39a-b) also correspond to different syntactic positions. Interestingly, the two distinct interpretations in (40) are possible with **KENDİ** in the same linear position. However, **KENDİ** in its emphatic function can also occupy different positions in the sentence (41), including the rightmost position (41c), which is ungrammatical for a traditional reflexive **KENDİ** as (42) shows (note also that emphatic **KENDİ** in (41) is optional):


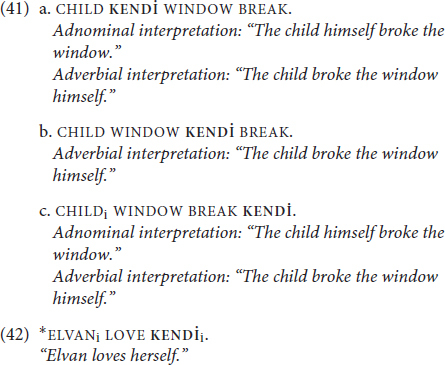


Moreover, as the translations for (41a) and (41c) indicate, emphatic **KENDİ** has both adnominal and adverbial interpretive possibilities in each of these possible positions.

However, Ahn identified other diagnostics that distinguish adnominal and adverbial emphatic anaphora, such as: denying the event, specificity, thematic roles, context-free acceptability, stative verbs, prosody and stress. Here, we will use three of Ahn’s diagnostics to probe emphatic usages of **KENDİ**: (i) denying the event, (ii) specificity, and (iii) thematic roles of the co-referent (research is ongoing, and we do not have enough data to conduct all the diagnostics suggested by Ahn (2010) at this stage).

Denying the adnominal emphatic requires denying that the focused referent was, in fact, the relevant event participant at all; in (43a), this is accomplished by asserting that someone else did the activity. Denying the adverbial emphatic, however, doesn’t mean denying that the individual did the thing, just that they did the thing alone or without help. The felicitous adverbial denial pattern is given in (43b).


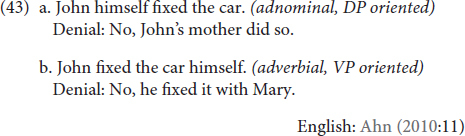


In (44), we see that *both* denials are felicitous with emphatic usages of **KENDİ** in each of the positions identified in (41)—that is, all three positions are apparently compatible with both adnominal and adverbial interpretations of **KENDİ**.


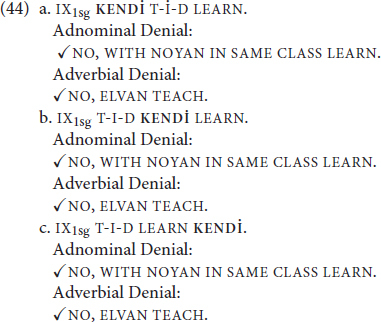


In addition to differences in deniability patterns, Ahn (2010) also observes that adnominal emphatic anaphors require a specific (but not necessarily definite) referent (45a,c), whereas no such restriction holds for adverbial emphatics (45b,d). “Specificity” here refers to the event participant being a unique entity as opposed to a generic one.


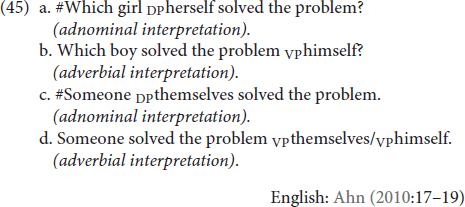


As for TİD, we again see a slightly different pattern. Non-specific referents—namely, WHICH CHILD (46) and SOMEONE (47)—are semantically compatible with the adverbial interpretation of emphatic **KENDİ**, as in English, but they are also compatible with adnominal interpretations. Here, too, these observations hold for **KENDİ** in multiple positions.


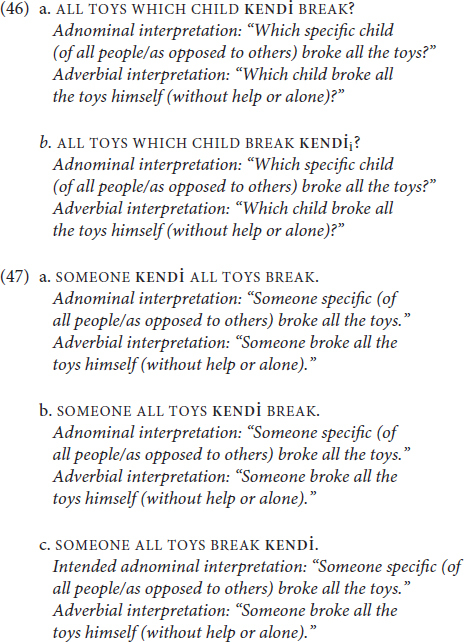


The third and final diagnostic that we will cover from Ahn (2010) concerns the thematic roles that are compatible with the emphatic anaphor. He observes that adverbial emphatic anaphors are only compatible with volitional and agentive subjects, whereas adnominal emphatic anaphors have no observed thematic role restriction. However, (48) shows not only that **KENDİ** is perfectly acceptable and grammatical with a non-volitional inanimate subject, but that it is compatible with an adverbial interpretation (EXTCL glosses an extension classifier sign):


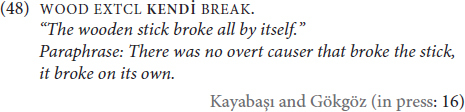


So far, our assessment of the emphatic usages of **KENDİ**shows us that they have more flexibility than (i) their traditional reflexive counterparts in TİD and (ii) their emphatic anaphor counterparts in English. However, it is important to note that the semantics and pragmatics of TİD is very understudied, as is the cross-linguistic typology of emphatic anaphors. Future investigation can investigate the source of these cross-linguistic differences and further assess if there are differences between adnominal and adverbial interpretations of **KENDİ**.

### Ambiguity Between the Emphatic and the Traditional Reflexive: Optional Argumenthood

In this section, we will explore cases where **KENDİ** can be ambiguous between a traditional reflexive and an emphatic anaphor. Such cases are possible when the predicate of a sentence allows for object drop or null objects and a potentially reflexive event. The predicate VOTE GIVE can take a DP (49a) or PP (49b) as an object, and it also can be used intransitively (49c):


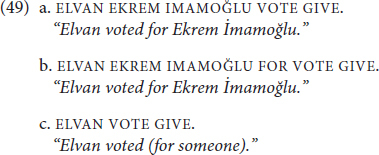


Thus, in (50), it is not clear whether **KENDİ** occupies an argument position as a traditional reflexive, or whether it’s an emphatic anaphor, and within the latter both adnominal and adverbial interpretations are possible.


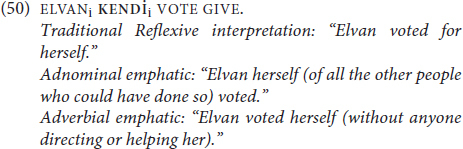


We assume here that this is a case of structural ambiguity: despite **KENDİ** surfacing in the same linear position, it occupies different positions in the sentential structure. The intended interpretations can be contextually disambiguated but they can also be structurally disambiguated^12^. Examples of structural disambiguation are illustrated in (51), where the presence of a preposition (51a) or a separate DP (51b) unambiguously express the intended transitive interpretation (recall from (49a-b) that VOTE GIVE can take its object as a DP or PP, which is why the FOR is “optional”).


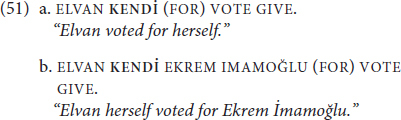


The above examples are cases where we see a given token of **KENDİ** that is compatible with different types of interpretations. However, there are also cases where we see multiple tokens of **KENDİ** within the same utterance, as in (52).


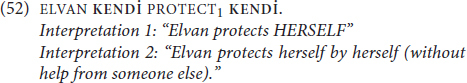


For the first interpretation of (52), it might be the case that the doubling of **KENDİ** functions in a similar way to focus doubling (Makaroğlu, 2012). The second interpretation, however, provides clear evidence that **KENDİ** can serve distinct functions, which can be combined within the same sentence.

## Conclusion and Future Directions

In this paper, we discussed the co-referential properties and the syntactic category of the sign **KENDİ** in TİD, which we argue can serve both a traditional reflexive and emphatic function. We have shown that **KENDİ** in TİD can function as a reflexive in the traditional sense and we have used syntactic typologies to classify it as a reflexive of the DP type. As a reflexive, **KENDİ**is subject to structural antecedence requirements. As a DP-type reflexive, **KENDİ** is able to serve functions outside of traditional reflexivity.

Importantly, this study lays the groundwork for further analyses of **KENDİ** as well as reflexivity in TİD and other sign languages in general. With respect to TİD, future research can expand our understanding of (i) the shared and different properties of the traditional reflexive and emphatic function of **KENDİ**, (ii) whether these properties are associated with distinct merge positions in the sentential structure, (iii) if logophoric usages of **KENDİ** are possible, (iv) non-manual characteristics of these distinct functions functions, and (v) potential language contact and bilingualism effects, among others. Moreover, future research can explore these issues in other signed languages, and further contribute to a cross-modal understanding of how co-reference is encoded.

## Data Availability Statement

The original contributions presented in the study are included in the article/supplementary material, further inquiries can be directed to the corresponding author/s.

## Ethics Statement

The studies involving human participants were reviewed and approved by Institutional Review Board of University of Michigan. Written informed consent for participation was not required for this study in accordance with the national legislation and the institutional requirements. Written informed consent was obtained.

## Author Contributions

DK conducted the fieldwork and drafted the manuscript, with critical revisions provided by NA. Both authors discussed the literature reviewed, designed the content and structure of the article, and approved the final version of the manuscript.

## Conflict of Interest

The authors declare that the research was conducted in the absence of any commercial or financial relationships that could be construed as a potential conflict of interest.

## Publisher’s Note

All claims expressed in this article are solely those of the authors and do not necessarily represent those of their affiliated organizations, or those of the publisher, the editors and the reviewers. Any product that may be evaluated in this article, or claim that may be made by its manufacturer, is not guaranteed or endorsed by the publisher.

## Footnotes

1 Koulidobrova (2009, 2011), Fischer and Johnson (2012), and Mathur (1996) observe that the reflexive pronoun “SELF” in American Sign Language also serves other functions. We discuss this phenomenon more in section “The Function of **KENDİ** Beyond Traditional Reflexivity”.

2 Note that the suffix noted here as *-un* is in fact {-(I)n} and becomes *-un* in this particular example due to vowel harmony (Göksel and Kerslake, 2004).

3 The subscripts a, b, c are used to express locus, and the subscripts i, j, k are used to express co-reference throughout the glosses. For the examples in which both locus and co-reference is marked, they are used as “a_i”.

5 See Pfau and Quer (2008) for a detailed discussion and analysis of manual and non-manual negation cliticization using data from Catalan Sign Language (LSC) and German Sign Language (DGS).

6 Fv: final vowel. The final vowel on verbs alternate based on the features of its object in Shona, and therefore marked in glosses the same way it was marked in the cited source. See Storoshenko (2009) for a more detailed explanation.

7 Handshape may serve as a kind of gender agreement marker in Japanese Sign Language, Taiwan Sign Language, and Korean Sign Language, but these too spatialize when marking verbs (Fischer, 1996; Zeshan, 2006).

8 İşsever and Makaroğlu (2017) observes sentences like (25b), where **KENDİ** co-occurs with a verb that is marked for reflexiv(ized) agreement, are ungrammatical, and account for it by the Anaphor-Agreement Effect. However, one should keep in mind that their data comes from signers from Ankara, and ours from İstanbul, indicating there might be dialectal variation in this. Note that Kimmelman (2009) also observes an anaphoric reflexive being accompanied by a verb marked by reflexive agreement in RSL.

9 Moreover, comparing (a-b) below, we see in (b) that the movement for the verb starts from a locus in neutral signing space and moves toward the signer’s body, which is being used as a stand in for non-first person (in addition to its use as shifted first person under role shift, Meir et al., 2007). a. IX_3sg_ IX_3sg 3_SUPPORT_3_   b. IX_3sg_ **KENDİ** SUPPORT_1_. *“He supports him.”             “He supports himself.”*

10 Dikyuva et al. (2017:204) observe that **KENDİ** is accompanied by a non-manual “op” mouth gesture when used as a possessor, and that it is a restricted to valuable and precious entities. Though we leave the “op” mouth gesture as a matter for future research, we do note here that our consultants did not share the intuition that possessive **KENDİ** is incompatible with ordinary noun possessees, as illustrated in (a-b). The reader is referred to Barker (1991) for a discussion of semantic issues like these in possessive structures, and to Abner (2013) for a discussion of these issues in ASL. a. NOYAN_i_a_ IX_a_ **KENDİ**_i_ BOOK_a_GIVE_1_. *“Noyan gave his own book to me.”* b. NOYAN_i_a_ IX_a_ **KENDİ**_i_ BOOK **KENDİ**_i_ STUDENT_b_ IX_b a_GIVE_b_ *“Noyan gave his own book to his own student.”*

11 See Ahn (2010:10) for a discussion of how the plain adverbial emphatic *himself* in (39b) compares to the *by himself* phrasal modifier.

12 As noted, that context can disambiguate **KENDİ** with no added structural cue necessary: *Context: There was an election in the university to be the department chair. Aslı_i_ and Meltem_j_ are candidates. Aslı_i_ is the former chair.* ASLI_i_ ONCE-MORE DEPARTMENT CHAIR BE WANT. IX_3sg_ **KENDİ**_i_ VOTE. IX_3sg_i_ MELTEM_j_ VOTE NOT. *“Aslı wants to be the department chair again. She voted for herself, didn’t vote for Meltem”.*
